# Endothelin ET_A_ receptors predominate in chronic thromboembolic pulmonary hypertension

**DOI:** 10.1016/j.lfs.2016.02.036

**Published:** 2016-08-15

**Authors:** Mark Southwood, Robert V. MacKenzie Ross, Rhoda E. Kuc, Guy Hagan, Karen K. Sheares, David P. Jenkins, Martin Goddard, Anthony P. Davenport, Joanna Pepke-Zaba

**Affiliations:** a Papworth Hospital, Cambridge, UK,; b Experimental Medicine and Therapeutics, University of Cambridge, Cambridge, UK; c Royal United Hospitals, Bath, UK

**Keywords:** Endothelin-1, Autoradiography, Immunocytochemistry, Chronic thromboembolic pulmonary hypertension, Pulmonary endarterectomy

## Abstract

**Aims:**

Endothelin-1 levels are raised in chronic thromboembolic pulmonary hypertension. Our aim in this study was to identify the presence of endothelin receptors in patients with CTEPH by analysing tissue removed at pulmonary endarterectomy.

**Main methods:**

Pulmonary endarterectomy tissue cross-sections were analysed using autoradiography with [^125^I]-ET-1 using ligands selective for ET_A_ or ET_B_ to determine sub-type distribution. The precise cellular localisation of ET_A_ and ET_B_ receptors was determined using selective antisera to both sub-types and compared with haematoxylin and eosin, Elastic Van Gieson and smooth muscle actin labelled sections.

**Key findings:**

Two patterns of ET-1 binding were found. In sections with frequent recanalised channels, ET-1 bound to the smooth muscle cells surrounding the channels. In sections where there was less organised thrombus with no obvious re-canalisation, minimal ET-1 binding was observed. Some contractile type smooth muscle cells not associated with recanalised channels and diffusely spread throughout the PEA material were associated with ET receptor antibody binding on immunohistochemistry. There was a greater expression of the ET_A_ receptor type in the specimens.

**Significance:**

The presence of ET-1 receptors in the chronic thrombus in proximal CTEPH suggests ET-1 could act not only on the distal vasculopathy in the unobstructed vessels but may also stimulate smooth muscle cell proliferation within chronic clot. The abundance of ET receptors within the tissue provides evidence that the ET pathway is involved in the pathology of chronic thrombus reorganisation leading to CTEPH providing a rationale for the repurposing of ET receptor antagonists in the treatment of this condition.

## 1 Introduction

At present there is a limited understanding of the factors responsible for failure of resolution of acute pulmonary embolism and the subsequent development of chronic thromboembolic pulmonary hypertension (CTEPH). The raised pulmonary vascular resistance (PVR) in CTEPH is described by a two compartment model [20]. In some regions there are thromboembolic occlusions of the vascular lumen and a series of associated changes including clot remodelling, collagen deposition and cellular hyperplasia. The ‘closed’ arterial tree distal to these obstructions is spared from exposure to high pressures. In other regions the ‘open’ arterial tree is exposed to high pressures and demonstrates pathological changes similar to those seen in pulmonary arterial hypertension (PAH); a distal vasculopathy with muscularisation of the distal precapillary arteries and intimal hyperplasia with medial hypertrophy of some larger pulmonary arteries [20]. Currently, CTEPH is the only form of pulmonary hypertension for which there is a potential cure; pulmonary endarterectomy (PEA), through the surgical removal of proximal chronic thromboembolic material [22].

Endothelin-1 (ET-1) is a potent vasoconstrictor. High levels of circulating ET-1 or its precursor big ET-1 have been demonstrated in patients with idiopathic PAH [9], [15], [29]. The ET pathway is considered an important part of the pathology of idiopathic PAH and ET receptor antagonists such as bosentan and ambrisentan [17], [18] and more recently macitentan [7] are used to try to slow progression. To date there is no ET antagonist licenced for use in CTEPH patients, although they are used ‘off-label’. The BENEFIT study did show a significant haemodynamic improvement with reduction in PVR, but no functional improvement with bosentan [11]. ET-1 acts via two receptors, ET_A_ and ET_B_, which have different effects upon pulmonary artery smooth muscle cells. The ET_A_ receptor activation results in vasoconstriction and smooth muscle cell proliferation. The ET_B_ receptor activation prevents apoptosis of smooth muscle cells and causes vasodilatation via nitric oxide stimulation. ET-1 is a potent vasoconstrictor but is also a promoter of pulmonary artery smooth muscle cell proliferation [8]. In agreement, Quarck et al. [5] found that pulmonary arterial smooth muscle cells isolated from patients with CTEPH have enhanced proliferative properties.

In patients with CTEPH, ET-1 levels are raised and have been shown to fall after PEA surgery [24]. After an acute pulmonary embolism there is obstruction of the pulmonary arteries by acute thrombus and elevated levels of ET-1 [28], [30]. Elevated levels of ET-1 have also been observed in air embolus in animal models [25], [26] and pretreatment with an ET antagonist ameliorated the haemodynamic change after acute pulmonary embolism [25]. In addition ET-1 is increased most in the muscularised pulmonary arteries [16]. Intriguingly in human coronary arteries we have previously shown that recanalisation of thrombus is characterised by formation of new vessels which show intense endothelial ET-like immunoreactivity with ET_A_ but not ET_B_ receptors on the smooth muscle of recanalised vessels [2]. Crucially, ET antagonists have been shown to block proliferation of smooth muscle in the intimal layer of vessels growing in organ culture [19]. The pathogenesis of CTEPH is complex but one question that arises is how much of the disease progression is driven by changes within the unresolved thrombus in parallel to the vasculopathy in the distal arterial bed. Despite many centres offering off-label use of ET receptor antagonist in the treatment of CTEPH, little is understood concerning the presence and underlying pattern distribution of ET receptors within PEA material. Here we identify ET receptors in PEA material and provide a rationale for the ET receptor antagonists for treatment of CTEPH.

## 2 Material and methods

### 2.1 Human tissue samples

Human tissues were obtained with informed consent from the Papworth Hospital Research Tissue Bank (REC 08/H0304/56) and local approval (REC 05/Q0104/142). Tissue specimens were collected from consecutive patients undergoing PEA surgery for analysis. Information was collected prospectively based on factors that were considered relevant to the interpretation of the histological appearance and any ET receptor pattern. This included the use and type of targeted therapy for pulmonary hypertension prior to surgery, haemodynamics from at the time of diagnosis, the intraoperative macroscopic surgical assessment of the type of chronic thromboembolic pulmonary arterial obstruction [12] and follow up post-operative haemodynamic measurements from assessment at least 3–6 months after PEA surgery. None of the patients had prior treatment with an ET receptor antagonist (to avoid the possible influence on ET receptor expression) [10].

**Fig. 1:**
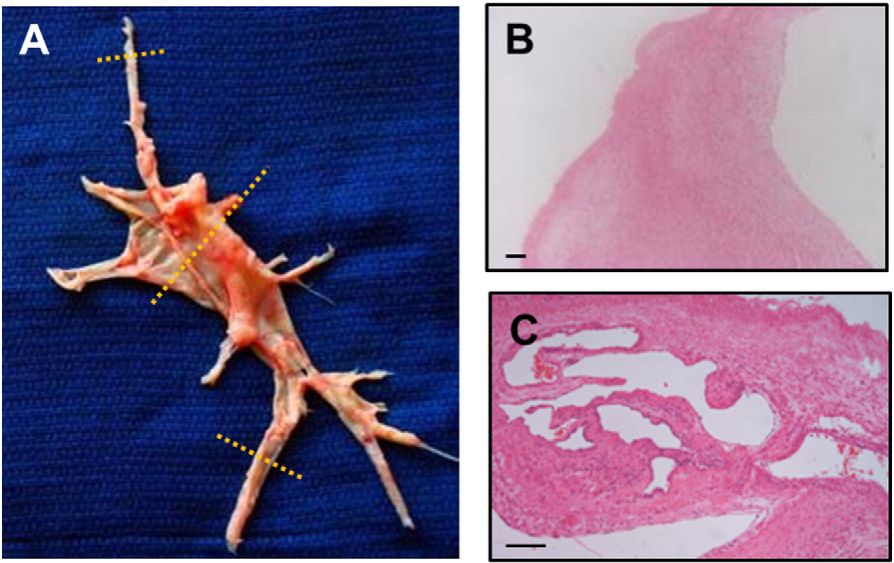
(A) Typical PEA material Example of a PEA surgical specimen from the left lung of a patient with CTEPH. Cross-sections taken from the central proximal and distal tail portions are indicated with dashed lines. (B), Typical findings of H&E stained proximal PEA material. (C) Typical findings of H&E stained distal PEA material, being highly organised with frequent neovessels (scale bars = 100 μm).

During the PEA surgery the tunica intima and a superficial layer of tunica media was removed along with the luminal contents. This tissue was then preserved in ice cold Krebs solution. Cross-sections were taken from the main endarterectomy cast, normally one from the most proximal portion and other samples from smaller, more distal portions of the PEA material [Fig. 1]. These samples were kept at − 80 °C and batched for autoradiographical analysis and confocal microscopy. Serial 10 μm sections were cut using a cryostat. Adjacent formalin fixed paraffin wax embedded blocks were taken for routine histological examination and immunohistochemistry. To determine the precise type of cell expressing the ET receptors, immunohistochemistry was performed to characterise the cell types that showed strong expression of the ET receptor in the ligand binding assays.

### 2.2 ***Autoradiography***

Autoradiography was carried out using established techniques [6], [13]. Briefly, sections were pre-incubated for 20 min at 23 °C in assay buffer (50 mM Hepes, 5 mM MgCl_2_, 0.3% BSA, pH 7.4) prior t, California) to determine the distribution of all ET receptors. ET receptor subtypes were visualised by incubating adjacent sections with [^125^I]-ET-1 (0.1 nM) alone to measure total binding, and in the presence of either 0.2 μM BQ3020 (to detect ET_A_) or with 0.1 μM BQ123 (to detect ET_B_). Receptor occupancy curves based on the known specificity of each compound were used to determine the concentration of each ligand. Non-specific binding was defined by incubating a further adjacent section with the radioligand in the presence of 1 μM unlabelled ET-1 (Peptide Institute, Osaka, Japan).

Following incubation the sections were washed (3 × 5 min) in ic*e*-cold Tris-HCl buffer (50 mM, pH 7.4), air dried and apposed, together with [^125^I]-ET-1 standards, to Kodak MR-1 autoradiography film for 3 days at room temperature. The films were processed and resulting autoradiograms photographed (Wild Heerbrugg microscope with Optim digital camera and Pixel Link OEM software).

### *2.3* Statistical tests

The degree of ET binding was assessed independently by two individuals and graded on the appearance of the autoradiogram alone. Broadly this was grouped into the following groups: none, < 25%, 25%–50%, 50%–75%, > 75%. Data were analysed using GraphPad Prism and difference between groups were assessed using a Student's *t-*test. A *p* value of < 0.05 was considered significant.

### 2.4 Histopathology, immunohistochemistry and confocal microscopy

Slides were prepared from cutting sections from each 4 μm wax embedded tissue block. After 48 h in the oven to melt the wax and dry the tissue, the slides were put in PT modules (Dako Ltd, UK) for 1 h at 98 °C for antigen retrieval. After washing (with PBS solution) the slides were exposed to hydrogen peroxidase 3% blocking solution (Dako Ltd, UK) before being washed again with PBS. The primary antibody incubations were all for one hour at room temperature. Immunolabelling was performed using polyclonal rabbit anti-human ET_A_ receptor antibody (ab84673, Abcam plc, Cambridge, UK); polyclonal rabbit anti-human ET_B_ receptor antibody C– terminal (ab84182, Abcam plc, Cambridge, UK), monoclonal mouse anti-human smooth muscle actin (Dako Ltd, UK) and monoclonal mouse anti-human CD31 (Dako Ltd, UK). Negative controls were incubated with EnVision Flex antibody diluent alone. Tissue sections were washed and incubated for 30 min with goat anti-mouse/rabbit secondary antibodies EnVision Flex HRP (Dako Ltd, UK), washed and labelled using Flex DAB + Chromogen (Dako Ltd, UK), washed and counter stained with Haematoxylin. The stained tissue sections were examined by a histopathologist with a special interest in pulmonary hypertension (MG). A brief description of the histological appearance for each section was made based on the degree of obstruction of the vessel lumen, the presence of smooth muscle cells within the pulmonary artery lumen, the presence of endothelialised and recanalised channels with distinct organisation of smooth muscle cells surrounding these new channels. Serial frozen sections were also used for co-localisation studies using polyclonal rabbit anti-human ET_A_ or polyclonal rabbit anti-human ET_B_ receptor antibody C-terminal (ab84182, Abcam plc, Cambridge, UK), incubated with goat anti-rabbit IgG conjugated Texas red (red). Cytoskeletal actin filaments [27] were labelled using alexa488 (green) conjugated phalloidin (Invitrogen, UK) and nuclei labelled using 4′,6-diamidino-2-phenylindole (DAPI; blue).

## 3 Results

Pulmonary endarterectomy specimens from 19 patients were collected; 14 male, 5 female. The mean age was 63 ± 9.7 years. One patient died in hospital and another patient did not return for post-operative follow-up. Table 1 shows the severity of the pulmonary hypertension and includes the surgical disease type (from the classification by [12]), haemodynamics at the time of initial diagnosis and any pre-operative drug treatment. The haemodynamic variables demonstrate reasonably severe pulmonary hypertension (pulmonary vascular resistance 763 ± 377 dyn·s·cm^− 5^). Ten patients had received pre-operative drug treatment with a phosphodiesterase V inhibitor, sildenafil, prior to PEA surgery. Mean pulmonary artery pressure (mPAP) was higher (*p* = 0.024) in the sildenafil treated group (50.90 ± 9.92 mm Hg) compared to patients not on therapy (39.33 ± 10.36 mm Hg). PVR was similar between both groups. Pre-operative drug treatment did not appear to have any significant effect on ET receptor expression (data not shown). The majority of cases were Jaimeson classification type 1 or type 2. A typical example of a PEA specimen is included in Fig. 1A.

§ = died following PEA surgery. # = did not return for follow-up.

### 3.1 Histopathology of PEA material

Forty three samples of tissue were taken for examination. Microscopically, the histological specimens were found to contain regions of fresh thrombus, intimal fibrosis with some degree of medial hyperplasia and organised thrombus in keeping with previous reports [4], [23]. We observed overall appearance, approximate vessel dimensions, the degree of luminal obstruction and the number of recanalised channels. Proximal PEA material was comprised predominantly of matrix-rich fibrous tissue with little evidence of recanalization (Fig. 1B). The approximate size of the native vessels examined ranged between about 40 mm and 5 mm in diameter. 6 sections were either fragments, long strips of tissue or had no discernible intimal layer. For the remaining thirty seven sections the degree of occlusion of the lumen of the native pulmonary artery was < 50% in 8 specimens, > 50% in 8 specimens, 100% occluded in 21 specimens. Unsurprisingly the smaller diameter tissue specimens displayed more intraluminal obstruction than the larger specimens. Fresh thrombus comprised fibrous tissue and loose fibromyxoid tissue with little evidence of organisation or matrix components. In more distal PEA material frequent recanalised channels were observed in many specimens, particularly evident in those of a smaller size (Fig. 1C). We next sought to the cell morphologies and to characterise the ET binding in the organising and non-organised PEA material.

### 3.2 Autoradiographical ET receptor binding in PEA material

**Fig. 2:**
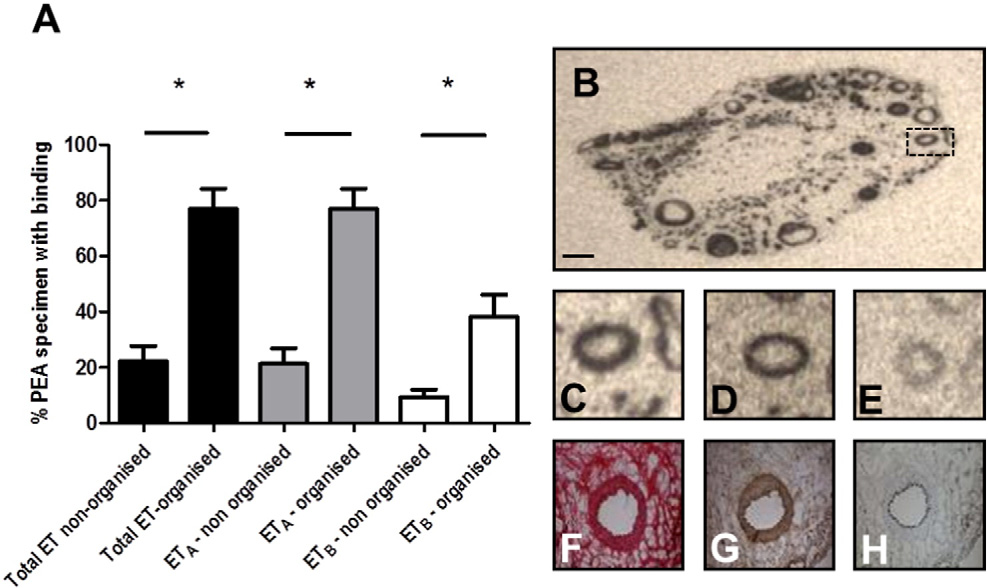
We estimated the % of the PEA specimen area to be either total ET, ETA or ETB positive in organised (predominantly distal) and non-organised (predominantly proximal PEA material. We found significantly higher levels of total ET and ETA receptors in organised PEA material compared to non-organised PEA material (A). A highly organised PEA specimen is also included in this figure where total ET (B and C), ETA (D), ETB (E) are summarised. Serial staining of adjacent sections with H&E (F), SMA (G) and CD31 (H) suggested that ETA receptors were predominantly colocalised to areas also displaying SMA immunostaining (scale bar = 0.5 mm).

Recent proximal thrombus with little evidence of neovascularization demonstrated no obvious pattern of expression for ET_A_ or ET_B_ receptors. In contrast, the autoradiograms showed ET binding was present in the majority of the distal pulmonary endarterectomy cross-sections. Specific ET receptor binding was observed in 32 of the 43 specimens. Autoradiography showed that ET_A_ predominated; all thirty two specimens express this sub-type. ET_B_ binding was detected in twenty eight specimens and was more limited within the organised thrombus where ET_B_ receptors co-localized with ET_A_ receptors in all but one case. The tissue specimens with 100% occlusion showed the greatest proportion of ET receptor binding. These specimens were generally smaller in diameter and displayed a greater number of recanalised channels and a higher degree thrombus organisation. We estimated the % of the PEA specimen area to be either total ET, ET_A_ or ET_B_ positive in organised (predominantly proximal) and non-organised (predominantly distal PEA material. We found significantly higher levels of total ET and ET_A_ receptors in organised PEA material compared to non-organised PEA material (Fig. 2A). A highly organised PEA specimen is also included in Fig. 2 where total ET (B and C), ET_A_ (D), ET_B_ (E) are summarised. Serial staining of adjacent sections with H&E (F), SMA (G) and CD31 (H) suggested that ET_B_ receptors were predominantly colocalised to areas also displaying SMA immunostaining.

### 3.3 Immunohistochemistry and confocal characterisation of ET receptors in PEA material

**Fig. 3:**
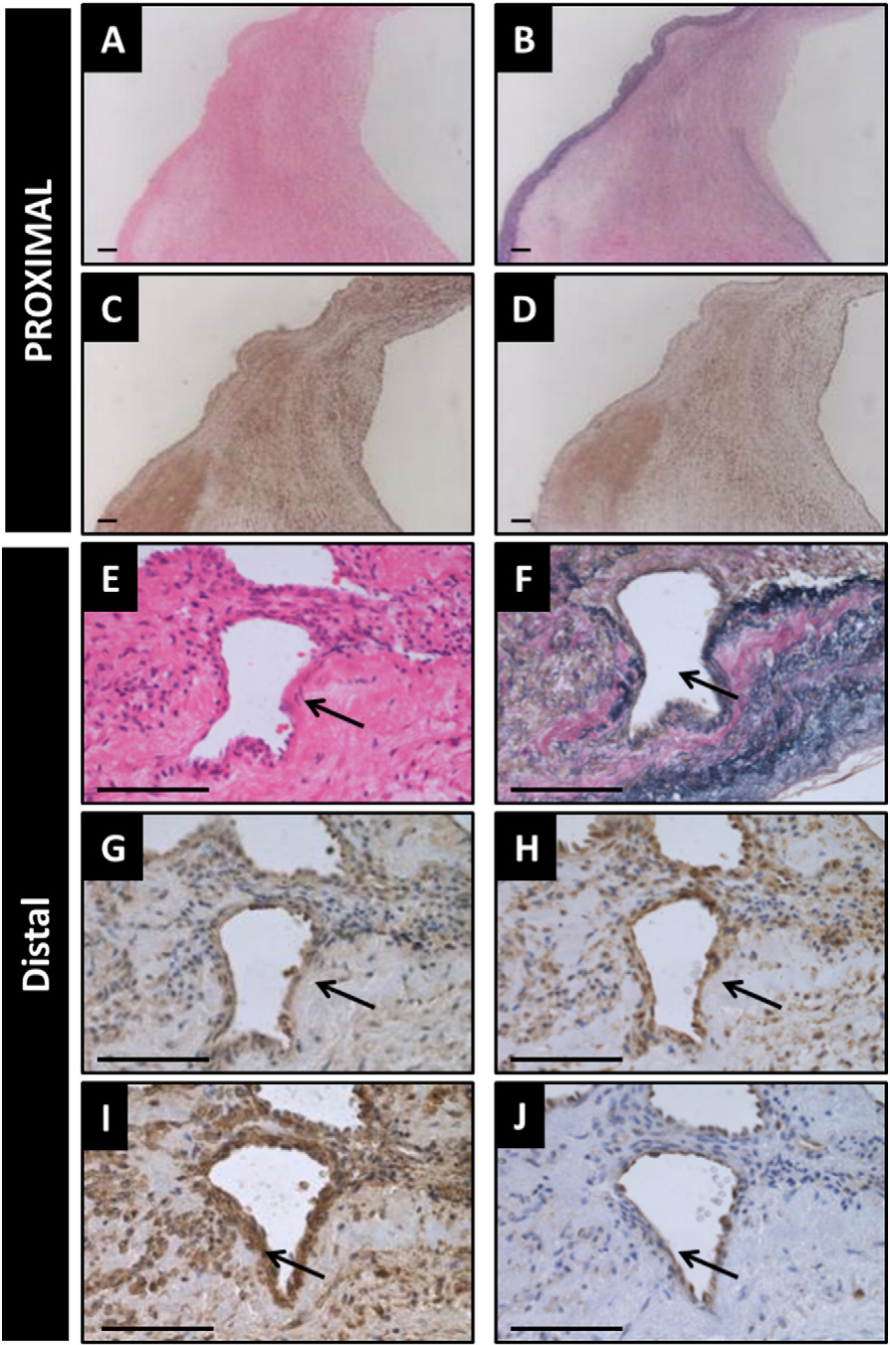
PEA histopathology and ET receptor expression. Representative images of the histopathology commonly observed in PEA material. Proximal PEA material are collagen rich (B) with little obvious pattern of (C) ETA or ETB (D) receptor expression. Distal PEA material tends to be more organised with the presence of frequent recanalised neovessels (E) surrounded by loose fibromyxoid tissue as demonstrated by H&E. An Elastic Van Gieson stain (F) demonstrates diffuse collagen (red) surrounded by elastin fibres (black). Immunostaining for ET_A_ (G) and ET_B_ (H) reveal vascular expression of both receptor types. Neovessels present in distal PEA material each recapitulating the histological architecture of an artery, having arranged contractile SMA-positive (fusiform) SMCs (I) and an endothelialised CD31 positive lumen (J). Arrows demonstrate an endothelial cell lined neovessel surrounded by organised SMCs. Scale bars in A–D = 100 μm. Scale bars in E–J = 50 μm.

To further understand the distribution of ET receptors in PEA material, we performed immunohistochemistry for ET_A_ and ET_B_ receptors with anti-human SMA-α (to label smooth muscle cells and myofibroblasts) and anti-human CD31 to identify endothelial cells. Basal levels of ET_A_ and ET_B_ expression were observed in regions populated with secretory SMCs distributed throughout the non- or loosely-organised regions of fibromyxoid connective tissue in the proximal material (Fig. 3A–D).

**Fig. 4:**
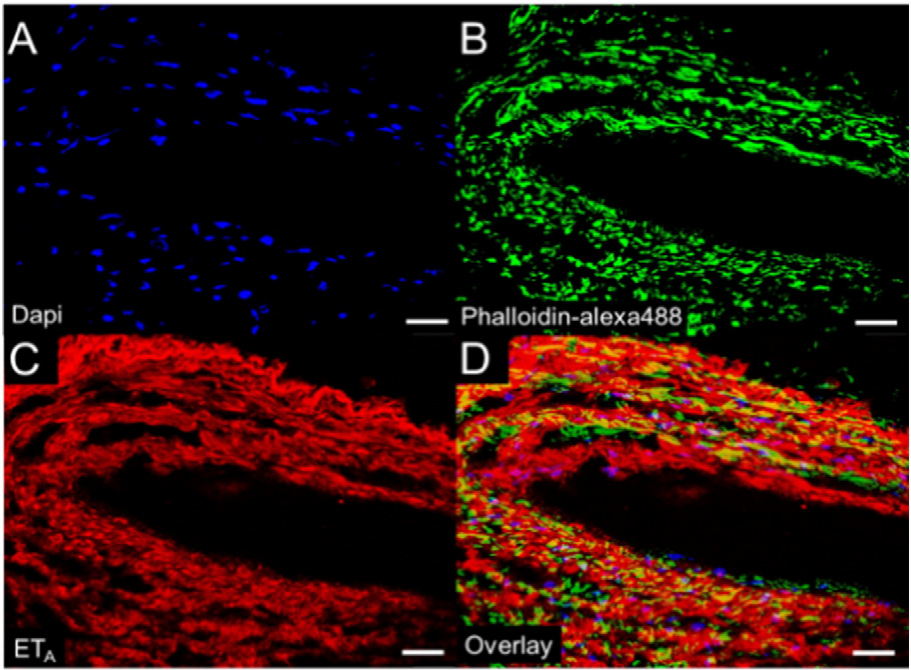
ET_A_ receptors are expressed by neovessels in PEA material. To confirm the vascular expression of ET_A_ in distal PEA material we undertook confocal microscopy. (A) Cell nuclei were labelled using 4′,6-diamidino-2-phenylindole (DAPI; blue). (B) Cytoskeletal actin filaments were labelled using alexa488 (green) conjugated phalloidin and (C) immunostained with polyclonal rabbit anti-human ET_A_ labelled with Texas red (red). Co-localisation studies confirmed strong ET_A_ expression to be associated with Actin filaments in the muscularised portion of the recanalised vessel (D). Scale bars = 25 μm.

The distal, smaller samples of PEA material commonly contained frequent recanalised neovessels surrounded by loose fibromyxoid tissue as demonstrated by H&E (Fig. 3E). An Elastic Van Gieson stain (Fig. 3F) demonstrates diffuse collagen (red) surrounded by elastin fibres (black). Immunohistochemistry revealed strong levels of ET_A_ (Fig. 3G) receptors and the presence of ET_B_ (Fig. 3H) to be expressed by the neovessels. Neovessel throughout the distal PEA material each recapitulated the histological architecture of an artery, being having a contractile ring of SMA-α positive fusiform myofibroblasts (Fig. 3I) with an endothelialised CD31-positive lumen (Fig. 3J). Co-localisation studies using confocal microscopy confirmed ET_A_ and ET_B_ expression to be associated with Actin filaments in the muscularised portion of the recanalised vessel (Fig. 4).

## 4 Discussion

CTEPH is caused by chronic thromboembolic obstruction of the pulmonary vasculature and the more distal portions of this residual material can completely occlude the lumen and have been shown to contain frequent newly formed vascular channels [1], [31]. Our results show that ET receptors are present within the luminal obstructions removed from pulmonary artery branches during PEA surgery for patients with CTEPH. To our knowledge this is the first time this has been shown using autoradiography. A crucial advantage of using autoradiography to identify ET receptors is that the receptor must be expressed on the cell surface and functionally viable for the radiolabelled ET ligand to bind. This provides mechanistic evidence for circulating ET to play a role in the organised thrombus present in the larger pulmonary vessels. This novel observation implies that ET receptor antagonists could act on the pulmonary circulation in CTEPH at the level of the organising thrombus as well as in the small-vessel arteriopathy. Analysis of the distribution of the ET receptors in the specimens shows that the larger less occluded pulmonary arteries (> 14 mm diameter) had fewer recanalised channels present and a lower expression of ET receptors, in contrast to the smaller specimens.

### 4.1 PEA specimen histology

The heterogeneous nature of the pulmonary endarterectomy specimens from our patient cohort is unsurprising. Macroscopically the surgical team could identify differences in the tissues removed at PEA surgery (using the classification from Jaimeson). To overcome the heterogeneity of the tissue, it would have been desirable to use a greater number of cross-sections from the same patient. This would have potentially allowed for a more systematic analysis of the surgical tissue based on anatomical location. This was not possible to perform as the bulk of the extracted tissue was required for histological analysis for the patient and could therefore not be sent away for autoradiography. The histopathological appearance of the cross-sections through our PEA specimens is consistent with previous studies [1], [4], [31] from pathological descriptions of CTEPH arising from post-mortem examinations, lung transplantation or from lung biopsies.

### 4.2 ET receptor distribution ET_A_ vs ET_B_

The ET_A_ receptor activation results in vasoconstriction and smooth muscle cell proliferation. The ET_B_ receptor is located in the endothelium and its activation prevents apoptosis of smooth muscle cells and causes vasodilatation via release of nitric oxide. The pattern of ET_A_ and ET_B_ receptor distribution in the lung has been previously demonstrated in animals [14] and humans [8]. In normal subject, congenital heart disease and idiopathic pulmonary hypertension subjects the larger calibre vessels showed predominant ET_A_ expression with a relative increase in the ET_B_ expression as the vessels narrow to 0.5-1 mm diameter (while remaining < 50% overall expression at this size). In the main pulmonary arteries > 90% of ET expression demonstrated in the medial layer is ET_A_ receptor. ET_B_ vascular expression is seen to a lesser degree in the small conduit arteries but at lower levels than seen for ET_A_. Within the rest of the lung ET_A_ expression is found in the parenchyma, airway smooth muscle and epithelial cells. ET_B_ is seen in high density in airway smooth muscle and lower levels in lung parenchyma and airway submucosal glands.

The results of our experiments on PEA specimens have shown that both receptors are expressed with more expression of ET_A_ receptors compared with ET_B_. This would fit with the size of our cross-sections indicating that they are conduit artery size where ET_A_ expression has been shown to be greater. The level of ET_B_ expression we have found is without question less than ET_A_ but in some specimens seemed greater than one might predict given data from congenital heart disease and idiopathic pulmonary arterial hypertension patients. Bauer et al. [3] have shown, in addition to elevated circulating plasma big ET-1 levels, elevated levels of ET_B_ mRNA with no change in ET_A_ mRNA in CTEPH specimens suggesting ET_B_ is upregulated.

Some similarities are thought to exist in the vascular remodelling seen both in CTEPH to those observed in IPAH [21]. The implication of this on our findings is that ET receptors will also be present in smaller surgically inaccessible vessel obstructions hence ET receptor antagonists might have some effect on the SMCs in those obstructions. A randomised placebo controlled trial of ET receptor antagonist in patients with inoperable chronic thromboembolic pulmonary hypertension and patients with persistent pulmonary hypertension after pulmonary endarterectomy surgery [11] has shown statistically significant improvement in PVR and cardiac output but it is not possible to know whether this was because of effects on the distal vasculopathy or proximal obstructions described by the ‘two compartment model of CTEPH’ [20].

## 5 Conclusion

This study demonstrates the presence of functionally viable ET receptors in the surgically extracted tissue from patients with CTEPH. The ET receptor was associated with smooth muscle cells, mainly the contractile phenotype of SMC that surrounds the recanalised channels and can be seen within the more organised chronic thrombus. Both the ET_A_ and ET_B_ receptors were found with the ET_A_ receptor showing more expression. This shows a potential role for ET receptors to influence both compartments in CTEPH and generates questions about how ET antagonists might influence the pathological development of the chronic thrombus in CTEPH.

Author contributions

RVMR, MS and MG carried out histology, data analysis, contributed to writing and manuscript review, REK carried out ligand binding and autoradiography, GH, KSS, DPJ identified and selected patient groups, APD and J P-K designed experiments, analysed data and contributed to writing and manuscript review.

## Conflicts of interest statement

Funded in part by an unrestricted educational grant to JP-Z from Actelion Pharmaceuticals, Basle, Switzerland. KKS has received educational support from Actelion, Bayer and GlaxoSmithKline to attend conferences. MS, RVMR, REK, GH, KKS, DPJ, MG and APD have no conflicts of interest.

## Figures and Tables

**Table 1:** Patient demographics.

Case	Surgical classification		Pre-PEA drug therapy	Pre-PEA mPAP (mm Hg)	Pre-PEA PVR (dyn·s·cm^− 5^)	Post-PEA mPAP (mm Hg)	Post-PEA PVR (dyn·s·cm^− 5^)
Right	Left
1	1	2	Sildenafil	55	794	26	218
2	1	1	None	50	816	17	146
3	1	1	None	47	908	25	156
4	2	3	Sildenafil	43	1162	37	549
5	1	2/3	None	29	315	25	202
6	2	3/4	Sildenafil	50	831	32	515
7	1	2	None	53	733	24	151
8	2	2	None	33	286	21	221
9	3	3	Sildenafil	74	1561	§	§
10	2	2	None	33	297	27	204
11	1	2	Sildenafil	43	491	38	304
12	2	2	Sildenafil	44	1053	19	163
13	1	1	Sildenafil	43	880	29	382
14	2	2	None	28	222	20	166
15	1	1	Sildenafil	47	888	43	545
16	1	2	Sildenafil	60	1274	50	702
17	1	1	Sildenafil	45	475	22	126
18	2	2	None	31	400	#	#
19	2	3	None	50	1504	42	1002
